# A Qualitative Study of the Development of Health Literacy Capacities of Participants Attending a Community-Based Cardiovascular Health Programme

**DOI:** 10.3390/ijerph15061157

**Published:** 2018-06-02

**Authors:** Verna B. McKenna, Jane Sixsmith, Margaret M. Barry

**Affiliations:** Health Promotion Research Centre, Discipline of Health Promotion, National University of Ireland Galway, H91 CF50 Galway, Ireland; jane.sixsmith@nuigalway.ie (J.S.); margaret.barry@nuigalway.ie (M.M.B.)

**Keywords:** health literacy, critical health literacy longitudinal qualitative research, determinants of health

## Abstract

Health literacy is a critical determinant of health, which can empower individuals and lead to engagement in collective health promotion action and is also a crucial component in the self-management of illness. The current study moves beyond a focus on functional health literacy and presents findings from a longitudinal qualitative (LQ) study consisting of three phases. This paper presents findings from the second phase of the study, which assessed the development of health literacy capacities of individuals attending a structured cardiovascular risk reduction programme in Ireland. The study objectives were to: explore perceptions of changes in interactions and information exchange within health consultations; identify the facilitators associated with changes in health literacy capacities; assess developments in engagement with broader contexts for health literacy capacities. A LQ study design was undertaken, which employed repeat interview methodology with 19 participants (aged 36–76 years) 12 weeks after beginning a structured cardiovascular risk reduction programme. Health literacy levels were assessed using the HLS-EU 47 item instrument in phase 1 (68% limited health literacy (HL), 32% adequate health literacy). A semi-structured interview guide, (informed by Sørensen’s conceptual model of health literacy), was used to explore the development of health literacy and to identify changes in knowledge, attitudes and experiences over time. Thematic analysis was used, informed by aspects of Saldaña’s framework for longitudinal qualitative data analysis. All participants reported having acquired increased understanding of issues relevant to their health and self-care. Participants described health literacy capacities that incorporate aspects of all levels of health literacy (functional, interactive and critical). Core themes were identified corresponding to changes in these levels: re-engagement with health information and increased understanding of risk and protective factors (changes in functional health literacy); changes in interactions with healthcare providers (HCP) (changes in interactive health literacy); enhanced psychological insights and understanding the broader determinants of health (changes in critical health literacy). Findings support the development of health literacy capacities across the functional, interactive and critical health literacy domains. Participants are capable of locating responsibility for health beyond the individual level and are making sense of knowledge within their own social contexts. Individuals, regardless of their initial health literacy levels, are capable of engaging with broader issues that can impact on their health and can be supported to develop these critical health literacy capacities.

## 1. Introduction

Health literacy is a critical determinant of health that can empower individuals and lead to engagement in collective health promotion action [1]. It is also a crucial component in the self-management of illness [2]. Regarded as an asset, it is seen as central to lifelong engagement with health, building cognitive and social skills as well as the motivation necessary to navigate healthcare systems, disease prevention and health promotion throughout the life course [3,4,5]. While there is increasing recognition of the need to support the development of individuals’ health literacy capacities and minimise environmental demands in both healthcare and broader societal contexts [6,7,8,9,10,11,12], there is still relatively little known about the barriers and facilitators in this process [13,14]. Research has highlighted the need to move beyond a focus on functional health literacy capacities in order to gain a greater insight into how the social and cultural context of individuals’ lives can facilitate the development of health literacy capacities, [12,15,16]. Some recent studies have examined this in adult-based educational settings [17,18]. This paper draws on the health literacy model developed by Sørensen et al. [5] to explore the different dimensions of health literacy within healthcare, disease prevention and health promotion settings. In this study, health literacy is defined as being’ linked to literacy and entails peoples’ knowledge, motivation and competencies to access, understand, appraise, and apply health information in order to make judgements and take decisions in everyday life concerning healthcare, disease prevention and health promotion to maintain or improve quality of life during the life course’ [5]. The current study addresses health literacy ‘capacities’, which refer specifically to the skills, abilities and potentialities of individuals to effectively access, understand, appraise and use information [6,19,20]. This definition aims to capture and embrace the depth of health literacy as an asset.

Increased insight is needed into how the development of health literacy capacity can be facilitated. The current study sets out to do this and presents findings from a longitudinal qualitative study, consisting of three phases (see Table 1 below), which examines developments in the health literacy of individuals over time. This study entails an in-depth qualitative exploration of the development of health literacy over time for a group of individuals managing their health and illness in the context of their everyday lives. There is a paucity of such longitudinal qualitative studies in the current health literacy literature.

This study makes an important contribution to the field of health literacy research as it is a qualitative in-depth and longitudinal study that follows individuals over time to examine developments in health literacy as they manage their health and illness in the context of their everyday lives. Findings from the first phase have been previously reported [22] and indicated a high level of limited health literacy for the population sample (65%). Both psychological factors, including perceptions of control and confidence in managing health, and structural factors such as access to health services and the impact of urban/rural environments, were found to impact on individuals’ use of health literacy capacities. Relationships with healthcare providers, mainly the general practitioner (GP), and the quality of that relationship, were also identified as being crucial in using health literacy skills. This paper presents on the second phase of the study, which assesses the development and perceptions of changes in health literacy capacities of individuals attending a structured cardiovascular risk reduction programme. The study aim was not to evaluate the impact of the programme per se. Rather, the programme was used as a ‘vehicle’ to engage individuals as they managed their health and illness over a 12-month period. The study objectives were to: explore perceptions of changes in interactions and information exchange within health consultations; identify the facilitators associated with changes in health literacy capacities; assess developments in engagement with broader contexts for health literacy capacities.

## 2. Methods

### 2.1. Study Design

This paper describes phase two of a longitudinal qualitative study design, which employed repeat interview methodology at three separate time points (see Table 1) to examine developments in the health literacy of individuals over time. Findings from time point 2 (T2) are outlined in this paper.

### 2.2. Participants

In this study, purposeful sampling was employed in order to select individuals attending a community-based structured cardiovascular risk reduction programme. Purposeful sampling is used in qualitative research to select individuals/sites for study that can purposefully inform an understanding of the research problem and central phenomenon in the study [23]. The sample in this study were selected in order to obtain the views and experiences of people with a range of risk factors for cardiovascular disease, as well as those with established disease (see Table 2). Twenty-six individuals were interviewed in phase one and nineteen of these were subsequently interviewed again at the 12-week point due to attrition of seven participants. The 12-week programme integrates the care of individuals with established heart disease and those at high multi-factorial risk of developing the disease, into a local community-based programme [24] that was originally developed at Imperial College London following the EUROACTION trial [25]. The programme is delivered by a multidisciplinary team comprising nurse specialists, dieticians and exercise specialists and incorporates weekly group exercise classes and educational workshops. The workshops address a range of topics including the risk factors for coronary heart disease and stroke, healthy eating, alcohol use, weight management, physical activity, stress management, food labels, maintaining change and cardiac medications. Participants also have weekly meetings with the multidisciplinary team [24]. Initial recruitment took place in conjunction with the programme nurse, who identified individuals who were cognitively able to participate and were able to communicate through the English language. A unique feature of the programme is that the partners of referred patients are also invited to complete the programme, and three partners were included in phase 2 of this study. Recruitment for this study took place between May 2014 and March 2015. 

#### 2.2.1. Profile of Study Participants

All of the participants (*n* = 19) had completed a 12-week cardiovascular disease (CVD) risk reduction programme and were referred through various pathways including general practice and hospital departments such as cardiology, stroke, and endocrinology. Participants had a variety of risk factors including hypertension and elevated cholesterol and many were also overweight or obese. In addition the majority of participants had experienced one or more of the following conditions: established heart disease (stents fitted), heart failure, type 2 diabetes, cardiac arrhythmia and stroke. Participant characteristics, including health literacy levels recorded at phase one, are summarised in Table 2. The raw scores of the general health literacy index are categorised to denote the following levels of health literacy: inadequate, problematic, sufficient and excellent health literacy. In this study these were further combined to yield scores for limited (inadequate and problematic) and adequate (sufficient and excellent) levels of health literacy. The limited level of health literacy reported here (68%) is significantly higher than levels reported in the overall European Health Literacy survey (47%) and in the Irish sample of the European survey (40%) [2,26]. These findings correspond with those for population subgroups with lower education and social class levels, and higher rates of disease and health service use, which is consistent with this participant profile.

#### 2.2.2. Data Collection Procedures

##### Interviews

Twenty-six individuals were interviewed in phase one and 19 of these were subsequently interviewed again at the 12-week point. Retention issues and attrition of participants is common in qualitative longitudinal studies [30,31,32]. In this study, attrition was attributed to a combination of issues including limited engagement with the risk-reduction programme on the part of some participants and illness factors that prevented programme completion.

Semi-structured interview guides were used to explore the development of health literacy and to identify changes in knowledge, attitudes and experiences over time (see Appendix A). The development of the interview guide was informed by Sørensen’s conceptual model of health literacy [5], in order to explore all the capacities associated with health literacy. Interview questions for phase 2, similar to phase 1, focussed on the areas of accessing, understanding, appraising and applying health information, and transcript data were initially categorised within these areas. In addition, questions were also included, in the form of probes, to explore further issues that had been identified in phase 1 (for example, concerns about upcoming treatment decisions). Saldaña’s framework [33] (as outlined in Table 3 below) was used to guide the analytic process and to structure the data analysis. The interview schedule was piloted prior to commencement of data collection with a small number of individuals attending the structured programme. Only minor changes were made to the sequencing of questions.

All interviews took place in a private room at the community-based programme building and were conducted by the first author (Verna B. McKenna). 

### 2.3. Data Analysis

All interviews were audio recorded digitally, transcribed verbatim and analysed using thematic analysis, which was facilitated through the use of N-vivo version 10 qualitative software. Qualitative validation criteria were applied in this study in line with established guidelines [34,35,36]. These included:

Credibility: Participants’ perspectives were reported as accurately as possible and the participants own voice used. Review and refinement of themes through a consensus among the three authors.
Triangulation: convergence sought among multiple sources of information (interview transcripts, memos, relevant theory and authors) to verify interview data and to develop themes. A level of member checking achieved where key issues and themes arising at time point 1 were reviewed with the participants at start of time point 2 interviews.
Transferability: Detailed accounts of the data and the context of data collection provided.
Descriptive validity: Multiple reading of the transcripts and listening to recordings in line with the methodology of thematic analysis.
Interpretive validity: Made clear through the use of the participants own voice alongside the meaning attributed by the researcher.
Theoretical validity: Findings clearly set out within relevant theory in the field of health literacy.
Researcher reflexivity: Preliminary analysis between time points allowed researcher to reflect on personal assumptions related to health literacy and social contexts.

The study used a hybrid approach of inductive and deductive coding and theme development, employing a thematic analysis methodology as advocated by Braun and Clarke [37] whereby core themes, subthemes and categories were identified. Aspects of Saldaña’s framework [33] (bolded in Table 3 above) were used in order to ensure that analysis captured the process of development and changes rather than presenting cross-sectional findings only [32,33]. By linking back to the previous dataset it was also possible to determine what changes or developments had occurred in terms of health literacy capacities (accessing, understanding, appraising and applying health information). The Saldaña framework [33] will be used more extensively for the overall longitudinal analysis of the entire dataset (time points 1–3). Preliminary analysis took place between interviews at time points 1 and 2 to allow reflexivity on the part of the researcher [38] as well as to focus on process and changes rather than on snapshots [32]. This preliminary analysis allowed the researcher to identify key issues that could then be returned to for further exploration in the second interview. This process was facilitated through the use of memos and field notes.

The analysis occurred both within each case and as a comparison between cases across the two time points. As such, the focus is not on gaining snapshots across time but to “ground the interviews in an exploration of processes and changes which look both forwards and backwards in time” [39] (p. 194), [40].

Credibility of findings was enhanced by returning to the original transcripts and through discussion with the other authors (Margaret M. Barry and Jane Sixsmith). A sample of transcripts were also read by Margaret M. Barry and initial codes, emerging themes and final themes reviewed and refined with both Margaret M. Barry and Jane Sixsmith through a negotiated consensus process.

### 2.4. Ethical Considerations

The study was independently reviewed and approved by the Research Ethics Committee, National University of Ireland, Galway in May 2013. All participants were provided with written and oral details of study participation and provided with written informed consent to participate in the study. Emphasis was placed on the voluntary nature of study participation, with the removal of all identifiers and assurance that all information would be anonymised. Due to the nature of longitudinal research, consent should be viewed as a process rather than an initial act [41]. In this study consent was requested from each individual at each time point. The Participant Information Sheet specifically set out that all participation was voluntary and that s/he was free to opt out of the study at any point.

## 3. Results

The interviews yielded rich data relating to developments in participants’ experiences of accessing, understanding, appraising and applying health information across various health contexts. Themes are set out in Table 4 and described in detail below. Themes are also linked to changes across the functional, interactive and critical domains of health literacy and highlighted below. Reference to the literature to support the changes across levels is also included where appropriate. Quotation labels are numbered by participant (P) and partner (PP) and also denote gender (M: Male; F: Female) and health literacy level (A: Adequate; L: Limited).

### 3.1. Re-Engagement with Health Information

Participants found it possible to re-engage with health information. This was attributed to *how* information was communicated, which was regarded as being central to facilitating developments across the health literacy capacities of accessing, understanding, appraising and applying health information. Involvement in the structured programme was perceived to be a positive experience overall for the participants. Despite the fact that the majority had been managing illness and/or risk factors for a number of years, individuals reported being able to find new ways of accessing, understanding, appraising and applying health information. This was particularly clear in relation to information pertaining to exercise, food and nutrition, and medication use. These findings are indicative of changes in functional health literacy whereby participants are increasing their ability to respond successfully to the communication of factual information on health risks [4,42].

In terms of the health information provided, comparisons were made to the more traditional or ‘boring sell’ (PP4FA). Although the information itself was not necessarily new, participants felt that it was the way it was delivered (more personalised and tailored health communication) that impacted positively on their ability to take it on board:

I would have read lots of stuff and you’d hear stuff on the radio about healthy eating and all the rest of it but by actually handing you the packet of cereal that you buy every week and saying if you really look at it; and so it was really, really pertinent to where we were at rather than saying you should eat more of this, eat more of that.

(PP4FA)

Participants, who at phase one had a recent diagnosis or illness event, were able to reflect back on the experience during the phase 2 interview. One participant described how she was now able to access information and reassurance regarding her husband’s stroke and recovery that had not been available to her in the hospital setting including the knowledge that it was safe to engage in exercise:

So we got an awful lot of information the first day we came—where the stroke was—they said it was in the front part of his brain—we didn’t know that from the hospital, you know. … they said it was alright for him to do it (exercise), which was more—do you know we never got that from the hospital really—they never told us like from once he came home.

(PP23FL)

Participants found that being able to communicate with programme staff helped them to understand and appraise information compared to reading information by themselves:

Because the books are great but then when you’ve somebody to talk you through it as well it’s good.

(P1FL)

Yes, I would, I’d find it easier alright now to kind of eliminate down and say well, yeah, now that is a thing that I really need to look into a bit more.

(P5FL)

### 3.2. Increased Understanding of Risk and Protective Factors

There were reported changes and developments in terms of understanding and awareness, which also led to application, i.e., how information was used. This in turn was linked to developments in engagement with and management of illness and risk factors. This was particularly evident in relation to food and nutrition, managing blood pressure and cholesterol levels, exercise, medication and treatments plans. These findings are also linked to changes in functional health literacy whereby participants are increasing their ability to act on the communication of factual information on health risks [4,42].

#### 3.2.1. Food and Nutrition

Although managing diet is a crucial component of treatment plans for cardiovascular disease, participants had limited previous knowledge of how to correctly read and understand food labels. This was a key aspect of new learning for participants that they were then able to apply to their everyday lives:

Like checking out labels and food products and what’s in some of the regular everyday foods that we just take for granted and don’t even give a second thought to. Maybe sweet foods or not foods that you’d imagine would have sugars and fats and stuff in them.

(P5FL)

They have it down as sodium or they have it down as something else and you’re like, these are all the hidden things like. Then like the sugar, the way they have it under syrup or corn syrup or under; it can be under different names, just little things.

(P1FL)

#### 3.2.2. Medication Use and Managing Side Effects

Although many participants were on long-term medication/treatment plans (such as medications for cholesterol and blood pressure), they were able to acquire new learning in relation to their use of medications as well as improved understanding regarding side effects:

Just that there are different medications for the blood pressure and you don’t have to stop at one; sometimes they combine two different things that you need, like two different tablets.

(P1FL)

This participant also reflected on how her childhood experiences had influenced her view on medication use, which, although entrenched, had now shifted towards a prevention first approach:

Like I think because my own mam and dad, they were on loads of medication so as kids it was like medication would fix you and I think since here it’s like why not prevent it before you get to the stage of medication …

(P1FL)

Participants in phase 1 had spoken about concerns regarding possible side effects of certain medications or treatment plans. They were now able to reflect on new learning regarding the possible side effects of different medications and how these could be managed. For others, this reinforced their opposition to certain medications:

I opted not to take (a statin) because I had read things about it… you know, I kind of feel I’ve gone from never taking anything to suddenly taking medication, you know… Yeah, I am always aware that I have responsibility but I will listen to research but I will also ask the question because I do, you know.

(P8FL)

Well I started off with having high cholesterol and it was 7 this time last year and we had that conversation about statins and I wouldn’t go on them and I went on my broccoli and my kale and it’s down now to, presently, to 5.5 so I’m happy the way it’s going. If I can keep bringing it down now it’ll be great.

(P12FL)

Others had seen positive changes in their medication regimens:

I’m off a lot of tablets I used to be on—a lot of my diabetic tablets that I was on have been more than halved.

(P13MA)

So it’s great that way and that way you can pass it on hopefully because I don’t have to take my blood pressure tablets any more.

(P1FL)

### 3.3. Changes in Interactions with HCP

Participants reported perceived changes in how they interacted with their GP. Participants identified increased confidence due to improved understanding of their conditions and/or having the reassurance of programme feedback to support them in their interactions with their GP. These findings reflect changes in the interactive or communicative level of health literacy whereby participants’ motivation and self-confidence to act on information was increased. One participant reported that she felt better able to communicate with her GP because she now has more knowledge about health issues such as blood pressure:

Just like when we were talking about blood pressure I was like, I know how it is. … Yeah, because I’d know more about it, I’d be able to say well this, that or the other. I’d be able to say no and isn’t this that and he’d be like, yeah, I’d be more comfortable about it because then I’d know what I’m talking about.

(P1FL)

Participants reported increased confidence in asking questions and felt that the credence of having attended a risk reduction programme supported their new found knowledge and helped in decision-making related to their treatment plans:

Probably because I’m not just dealing with my own GP, there are other influences and I can kind of, and other people that I’ve got contact with here and I suppose that can sort of say well this has been said and what do you think? That to me I think is important because you know sometimes you do feel you’re at the mercy sometimes of, you know, if you’re just dealing with one person. …. I mean, that was the good thing of this, I kind of feel now I’ve got more than one area to pull on.

(P8FL)

I suppose the reinforcement by the people here from what my GP was saying, you know, when you’re dealing with one person I was able to kind of say to him ‘can you leave me another month?’ But they were very definite here, ‘oh you actually need your medications increased.’ So it was reinforced in two places. It was very definite, and I kind of knew they were right, you know.

(P21FL)

Participants also commented on the contrast between the busy GP practice and the access to staff on the programme particularly for emotional support:

GPs are very busy, you know, you have so many things when you go in I feel of the medical type … but really you are conscious that they are so busy and there is so many more waiting to come in, that, no, you know, I don’t think it’s a good place to sort out feelings.

(P21FL)

Another participant acknowledged that her communication/interactions with GP were more relaxed as she was less anxious now regarding her health condition:

I was probably more relaxed this time than I would have been normally and I probably wouldn’t, I probably allowed my GP to just get on with the job herself as opposed to interrogating the poor woman … and at the same time, for example, I suppose I would have been less anxious in many ways; that would be an indication of me being more willing to trust that things are probably alright but no harm to check things out.

(P2FA)

### 3.4. Enhanced Psychological Insights

This theme refers to individuals’ increased insight regarding personal control limits and opportunities and also encompassed the areas of self-efficacy, emotional issues and facilitators of motivation. Similar to the findings from interviews in phase one, this theme was important in terms of its impact on individuals’ abilities to utilise their health literacy capacities to their fullest potential. Having the potential to fully engage with and use health information became more possible through gaining an increased understanding of their own situation. These findings also show that participants are becoming more empowered as they navigate illness and health management and are increasingly using of critical health literacy skills in attempting to control aspects of personal and social health determinants.

#### 3.4.1. Perceptions of Control and Self-Efficacy

Participants were able to reflect on developments in terms of the control they felt over their situations. One participant reflected on earlier challenges:

Everything was kind of against me. I remember at one of the meetings now alright and what they kind of said to me well at least like, you’re not in a great place but at least if you’re doing something about it, you know, you should feel that bit better in yourself. You’re trying to improve some of the situation anyway.

(P5FL)

This participant also made the comparison to what she was then able to achieve:

Maybe with them telling you and explaining to you that I suppose every point you come down in something or every month you’re doing something that it sort of helps you along the way.

(P5FL)

Another participant reflected on the importance of developing a greater understanding of health issues and having the time or space to pay attention to them:

You see, I suppose for myself, you know, when you’re busy, you’re working you don’t, I just didn’t think about my health, you know. I’m realising because I’ve had a lot go on in the last while, if I want to live healthily and I suppose now too when I’m retired; I think when you’re working sometimes you don’t have so much control but now that I’ve retired I think my attitude has changed.

(P8FL)

Another participant reflected on the role of stress and learning how to deal with it to have more control over health issues:

I let stress develop, it was like a cancer, it was eating away at me. So in the last few weeks I’ve started letting it go completely over my head, positive things have come out of it, you know. I’m totally relaxed, totally chilled out, which I wasn’t, and maybe that’s why my blood pressure was away, you know.

(P13MA)

Some participants also demonstrated a more comprehensive understanding of their situations and the factors that determine their health:

I can control my amount of exercise and diet, certain things you can control, but there are certain things you can’t in life. You know like your situation. Or if you wanted to go and live somewhere else, or live alone, or not having the stress of this that or the other, then there is some things you can’t control, you know, financially and that kind of thing.

(P21FL)

Developments in self-efficacy linked to exercise performance were also apparent. This was most evident for participants with mobility and illness concerns in phase 1. These participants felt reassured that exercise was both safe and possible:

I never was in a gym, I never used a gym before, or exercised with other people, or that kind of thing—I never realised how good you can feel after! I didn’t know that! I feel a bit braver. It made me feel, you know, that I could do it, and it’s nice to exercise.

(P21FL)

I feel that I have enough information about my illness, my sickness, I wouldn’t call it an illness either, but my health, that I feel I’m in safe hands. And if I reduced my chances of getting a stroke by 20 percent in 12 weeks, what can I do in another [12 weeks]? Like, they wanted to get my blood, my heartbeat up to a certain thing, and they’ve done that.

(P20FA)

#### 3.4.2. Anxiety and Fears

While participants had previously referred to the impact of anxiety on dealing with illness, they were now able to reflect on how these fears had been assuaged.

One participant spoke about her fears following her husband’s stroke and how this impacted her sense of control:

How do I put it? Like when the stroke came I didn’t know what I knew anymore, or what, because he was such an unlikely candidate of getting a stroke.

(PP23FL)

Participants also reflected on overcoming fears about illness:

So I just, I think I have a kind of a, I feel more confident that I’m not as bad as I thought I was, and that I know like that I can live a good life. I can look forward, I look forward now like to a better kind of a life for myself.

(P20FA)

The symptoms that I presented with, were sort of symptoms that would be relevant to a mini stroke. And that sort of made me afraid. That if I take a long journey in the car or should, should I do this, should I do that, will I get more symptoms, will I get a stroke? That, it knocked my confidence.

(P15FA)

This participant was now able to reflect with a more positive outlook, which she attributed to staff reassurance:

And they were all (saying) you know that might never happen again and your blood pressure is being monitored and your heart checks and cholesterol and diet … it takes a while to readjust … I’m smiling now and all that, but it did throw me … But I’ve come out the other end now and I’m ok.

(P15FA)

One participant with heart failure reflected on the fact that she was less fearful about taking part in activities:

And doing a bit more. But I’m not as nervous now about, say, taking off and doing more things by myself. Just up to the gym and keep doing it, and I knew that I could then, you know.

(P21FL)

### 3.5. Understanding of Broader Determinants of Health

Participants demonstrated an increased ability to reflect on external factors that can impact on health. These were related to physical contexts, such as walkability issues and access to cycle lanes; health and public policies, e.g., in relation to childhood obesity, affordability of medicines; and legislation in relation to food labelling standards. These findings can also be related to changes in critical health literacy, as participants are critically analysing health information and using this in an attempt to exert greater control over personal and social health determinants [4,42].

#### 3.5.1. Physical Contexts

##### Walking—Access and Road Safety Issues

Similar to the phase one findings, rural-dwelling participants were most critical of the lack of safe places to walk:

Just somewhere for people to be able to go out walking that’s safe, just a little footpath just for maybe three miles say and then everybody can go, because like it’s there, it’s safe. Women can bring their buggies, kids can go on bikes.

(P1FL)

This participant went on to make a comparison between two different areas and the impact of having safe walkways:

So like there’s loads of walkways and people are more motivated up there. You look out the window, every three seconds there’s somebody walking by; always movement, people are running or cycling or something but back in our place all you see is cars. It’s a different place … It would be great for everywhere because I know, say cities have it all but out in the country there’s really nothing.

(P1FL)

Similarly, from another participant:

Paths would make lives better for people, a lot easier. So if you were walking with a child in a pram you just couldn’t do it you know. And I mean an elderly person, my mother has one of those wheelers, she can’t walk. Those little things would make life a lot easier you know.

(P8FL)

Others, living in more urban areas appreciated access to scenic walkways and green spaces and not having to rely on a car:

But the effort of getting into a car and driving, you just won’t do that, whereas where we are we don’t have that excuse. We do not need to get into the car to go to somewhere specific to do exercise.

(P4FA)

I actually love where I live, in that I’m beside a big green, I’m beside a big park, so I can walk, in five minutes from my house I can walk in a big field, and it’s half a mile we’ll say one way and the other, I can have a half mile to a mile walk. And that really is important to me. Or I can go down to a beach. And that is really a lifeline for me, being able to live where I’m living. So I think that really has an impact on your health, if you have somewhere nice to walk, I think, it’s really important.

(P21FL)

##### Cycling

Participants who regularly cycled commented on the lack of a completed cycling infrastructure, which impacted on safety:

Once you go outside the city area back on the country roads like I’d be nervous enough now cycling. It would make it safer. Because you are taking your life in your hands if you get up on a bike…the traffic is too busy. So from that point of view I would like to see something being done.

(P16ML)

##### Food Culture/Food Manufacturing

A key learning in relation to food and nutrition was reading and understanding food labels, which led to reflection on broader issues such as the regulation of food labelling:

I think they’d want to get that system brought in for all food manufacturers. But there would be too much opposition from the ones who have the bad food. So there’s a stalemate there. So it’s up to the government to bring it in by law and that’s the only way it can happen. If they bring it in by law then they’re compelled to put that red label on and put their product, whether it’s red, amber or green.

(P3ML)

Another participant commented on the role of policy with regard to childhood obesity.

I would be very bothered about this new idea in the schools that they’ve a ‘no running’ policy now in 1 in 20 schools in the country. I think that’s where it needs to start with the health. Now I know they do some of these sports things and that, but there should be an awful lot more. They’re talking about having a sugar tax but then on the soft drinks and that but I think there should be a tax like cigarettes, you know, because it’s ridiculous.

(PP4FA)

##### Role of Government

One participant had taken part in a lobbying exercise to get the government to consider the provision of a medical card (free primary care access) for people with certain chronic diseases:

So I sent that in and wrote all my own views on it and the list of complaints that I have, all the heart problems I have, the liver and so on. And then the list of all the drugs I have. In other words I pay the maximum of, it’s over 1700 a year even with the allowance the government make, that’s what it works out at. And I’m retired so you know I’d appreciate it if the government were to do that; give me a medical card for the drugs. So that’s the one way I can appeal to the government.

(P3ML)

Another commented on a lack of long-term health promotion planning at government level.

And the other thing is that they’re putting in a whole load of wind farms, which is fine and I’m in favour of wind farms and alternative energy, but our carbon footprint would go way down if we actually cycled in the places that we can cycle. So I think there’s very little joined-up thinking in terms of health promotion.

(P2FA)

##### Sharing Information

Some participants were actively sharing health information with extended family/friends and some were actively trying to change the health practices of others:

So there’s little bits here and there, even just me in the class here; I’m passing it along because there’s 13 of us so I’m passing it on to them and their wives and their kids. So there’s a whole bundle of people out there that’s getting the information as well. Even the booklets that I get here I pass them on there and they’re all reading them. Some things they might change, some they mightn’t. You know that they are trying themselves as well because like a few of us started doing a few changes and you see them doing it as well; even like there’s a few of my brothers and they’ve given up salt altogether.

(P1FL)

I do especially say to immediate family. I did, I spoke to my niece about it, I spoke to my sister. I’m very much a kind of pass it on, I think that’s important. The more people who know, you know … Passing it on, kind of giving people, say my sister, both sisters actually and also my sister’s partner because he had a stent put in; I suppose I rang him then just this week and said are you aware that you really should have a blood test on a regular basis; well he was but he hadn’t, so that kind of thing.

(P8FL)

Participants recognised that dietary changes would also have a positive impact on other family members such as children and grandchildren.

I’ve a young lad there, he’s seven, he comes in [saying,] ‘Granddad, I’m starving.’ I say, ‘go to the fridge there and get something for yourself.’ And he’ll pick the fruit and he’ll eat it, whereas if it wasn’t there he’d be looking for Tayto (crisps) or something like that.

(P16ML)

But there isn’t as much around for them [grandchildren], there’s no biscuits around or anything like that. Yeah. And I thought first that we’d try it—I thought it wouldn’t last—but now it’s nearly normal, do you know, that we don’t have it.

(PP23FL)

## 4. Discussion

The overall aim of this study was to explore developments in the use of health literacy skills for individuals in the context of managing risk factors for CVD. The findings are consistent with an approach that goes beyond the functional aspects of health literacy to capture broader social contexts [43]. The findings have generated important insights into factors that support developments for health literacy capacities and indicate that individuals with varying levels of health literacy can engage with self-management. Findings support the development of health literacy capacities across the functional, interactive and critical health literacy domains and engagement with health knowledge that goes beyond personal health management to the social determinants of health [5,42]. 

Although many participants had been managing risk factors and/or conditions for some time, they found that it was possible to re-engage with information that was presented in a very practical and relevant format and tailored to them. In terms of managing lifestyle factors associated with illnesses such as diabetes, CVD and stroke, having an understanding of the importance of good nutrition and diet is crucial, as well as the ability to apply that information [44]. Participants learnt how to read and interpret food labels and were able to apply this information to their everyday lives. This allows for a greater sense of control over self-care behaviours and participants, therefore, experienced changes in how they perceived control and self-efficacy in relation to managing their risk factors. These findings indicate that participants are becoming more empowered and support the conceptualisation of health literacy as an instrument in the empowerment process [45]. Other studies [46] have examined specifically the role of self-efficacy and health literacy in improving health outcomes and also advocate the need for self-management programmes to promote the development of self-efficacy. Numerous studies have postulated the links between self-efficacy, health literacy and self-care behaviours [8,47,48]. Work by Lee et al. [48] suggests that studies focusing only on the functional aspects of health literacy may not reflect the relevance of self-efficacy. Therefore, interventions need to also focus on the communicative and critical aspects of health literacy [7,48,49]. A recently developed health literacy communication training for healthcare professionals has also emphasised the inclusion of skills to enhance both interactive (shared decision-making) and critical health (enabling self-management) skills of healthcare providers [50]. Participants in this study described health literacy experiences that incorporate aspects of both communicative/interactive health literacy (i.e., the ability to extract meaning from different sources of information and share the information) and critical health literacy experiences (i.e., the ability to critically analyse information and apply it to decision-making process). Findings in the current study indicate that participants also experienced enhanced self-efficacy in managing their health regardless of their level of health literacy at the start of programme (i.e., whether adequate or limited). This is an important finding as it may indicate that persons with limited health literacy can engage well on structured programmes and develop their health literacy at all levels as a result.

Similar to findings from the Skilled for Health study [51], there was evidence that participants where actively ‘cascading’ or sharing their newly acquired information with their family and wider community. Edwards, Wood, Davies and Edwards, [52] in their study used the term *health literacy mediators* to describe individuals who passed on their health literacy skills. This is linked to distributed health literacy though in this instance it is about supporting others to become more health literate (rather than being supported).

Findings in relation to anxiety provide further support of findings from time point 1 [22], which highlighted the impact of illness-related anxiety and fears on the ability to fully utilise health literacy capacities. In the current study, participants reported how their concerns had been alleviated and they were thus able to engage with and utilise health information more effectively. This concurs with work by Dunn, Margaritis and Anderson [13], which emphasised the role of the HCP in the provision of social support and reduction of anxiety for patients with CVD. Further, they also point out that patients are then in a better position to be able to take on board and have a better understanding of their condition. Morgan et al. [53] also stress the need for self-management support to include the broader contexts of an individual’s life so that management is not constrained within a narrow disease control approach [53]. Findings in this study support a view of participants, not as a patients but as active participants in their own health management, and more akin to being ‘citizens in relation to the health promotion efforts in the community, the work place, the educational system, the political arena and the market place’ [5] (p. 13).

Participants in this study participated in a community-based risk reduction programme, which provided them with the opportunity to make comparisons between typical healthcare encounters and those at the programme. There were many positives associated with how information was imparted; the group effect (‘collective efficacy’ as described by Bandura [54]), the accessibility and approachability of the staff; the time of staff; emotional support; social support; motivation etc. All of these factors would seem to be conducive to promoting effective self-management [53]. The programme also offered the opportunity to become more familiar with medication regimens and treatment plans including being aware of when and why changes to these were made. Overall, participants noted positive developments in their interactions with HCPs. These were attributed to having increased knowledge and understanding of one’s condition as well as a sense of increased confidence regarding communication with the HCP.

All participants reported having acquired increased understanding of issues relevant to their self-care. Findings indicated that participants had a new level of engagement with the issues as they often were exposed to a new perspective or information presented in a new way.

One of the most important findings was in relation to participants’ understanding of medications and side effects. It was noteworthy that participants continued to question the use of statins and continued to favour lifestyle approaches (where benefits seen). This understanding can be attributed to a combination of knowledge acquired both through the programme and through their own information-seeking.

An important finding in the current study is the engagement of participants with broader issues that can impact on health and the contextualisation of health issues. In terms of health literacy this is a shift towards critical health literacy whereby individuals start to address the demands and complexities of their social contexts [12]. These findings compare favourably to those reported by Rowlands et al. [16] where social determinants, social activity and the local community were viewed to influence the translation of knowledge into health behaviours. Participants were able to reflect on how physical and structural factors can directly affect one’s health and wellbeing. Participants’ responses also indicated that they are capable of locating responsibility (for health) beyond the individual level [55] and are making sense of knowledge within their own ‘social space’ [43] within their own family and community environments. This indicates that developments in health literacy have moved beyond the acquisition of knowledge to also use health literacy capacities as a resource for engaging in health at the community level [43,55]. The community-based programme offers the potential to develop critical health literacy and lends support to community-based programmes for the management of chronic illness. The potential to develop critical health literacy capacities is important. Beginning with having an increased insight into the limits and opportunities of one’s personal control, it is evident that there is potential to develop this further as an important element of self-management. Future research could build on the value of the qualitative longitudinal approach and incorporate more of a participative methodology whereby individuals could map and elucidate the facilitators and barriers in the empowerment process as health issues are managed. It would also be useful to examine this approach with individuals outside of access to a structured programme so that comparisons between organisation/system demands on health literacy could be made.

### 4.1. Strengths

A strength of the current study is the qualitative perspective, which allows for a more in-depth examination of the development of health literacy capacities from the perspective of individual study participants. The longitudinal aspect of this study also allows us to identify the types of factors that can contribute to the positive development of health literacy for individuals over time. It is also possible to see that developments occurred regardless of initial health literacy levels. It is important that these developments are explored outside of the clinical encounter so that aspects of social and community contexts, where health and illness management and health promotion activities frequently occur, are also addressed.

### 4.2. Limitations

The relatively small sample and the attrition of study participants from phase 1 to phase 2 is an important study limitation. Attrition is a frequently reported issue in longitudinal studies, which in the case of this study resulted mainly from the illness experiences of participants and limited programme engagement in a small number of cases.

Only preliminary analysis was possible between interviews at time one and the completion of time 2 interviews due to time constraints and resource limitations. However, the keeping of memos and field notes following all interviews assisted in the overall analysis process. This study is focussed on a specific population sample who attended a risk reduction programme. It is possible that some of the positive effects in relation to the development of health literacy capacities evident at the completion of phase 2 are due in part to the effects of programme participation. The fact that participants had just completed the 12-week programme is likely to have influenced their perceptions regarding positive outcomes.

## 5. Conclusions

Study findings demonstrate that, overall, participants have become more empowered in managing their health and self-care. Developments in terms of perceived confidence and self-efficacy were apparent, which in turn impacted on positive relations with HCPs. Crucially participants have also demonstrated an increased ability to critically reflect on social and environmental issues that can support or impede their health opportunities. These findings support the idea that health literacy should be regarded as context and content specific and that critical health literacy can be achieved in the absence of high functional health literacy skills [56,57]. Findings also demonstrated that individuals have the capacity to acquire new insights and perspectives in managing health issues, even where the illness is not new. This can mitigate against resignation regarding long-term treatment plans and the promotion of their own health in a more active manner.

## Figures and Tables

**Table 1:** Overview of timeline, sample and methods for overall longitudinal qualitative study.

Time Points	Sample	Methods
Phase 1: (Baseline: Beginning of programme)	26 Participants	HLS-EU survey and interview completed [21]
Phase 2: (End of programme @12 weeks)	19 Participants	Interview completed
Phase 3: (One-year follow-up @12 months)	17 Participants	HLS-EU survey and interview completed [21]

**Table 2:** Profile of study participants.

Participants (*n*)	19
Gender	*n*, %
Male	8 (42%)
Female	11 (58%)
Age (mean, range)	61 (36–76)
Education: highest level attained to date	*n*, %
Primary School	4 (21%)
Secondary School	10 (53%)
Diploma/certificate/Primary degree/postgraduate	5 (26%)
Social class ^1^	*n*, %
I–II (High)	6 (32%)
III–IV (Medium)	1 (5%)
V–VII (Low)	12 (63%)
General health literacy level from HLS-EU measure ^2^	*n*, %
Limited	13 (68%)
Adequate	6 (32%)
Length of time with risk factors/illness	*n*, %
6–9 months	7 (37%)
More than 1 year	12 (63%)
Health service access	*n*, %
Private health insurance	10 (53%)
Medical card only ^3^	7 (37%)
Private AND medical card	2 (10%)

^1^ [27]; ^2^ [21] ^3^ A medical card allows access to GP services, community health services, dental services, prescription medicines and hospital care free of charge under the General Medical Services Scheme for sub-groups of the population based on income levels/specific medical conditions [28,29].

**Table 3:** Saldaña framework [33] (aspects used in this analysis are bolded).

Framing Questions (5)
**What is different from one round of data to the next?**
When do changes occur?
**What contextual and intervening conditions appear to influence and affect participant changes over time?**
What are the dynamics of participant changes over time?
**What preliminary assertions about participant changes can be made as the data analysis progresses?**
Descriptive Qs (7)
**What increases/emerges over time?**
What is cumulative?
What kinds of surges/epiphanies occur?
What decreases/ceases over time?
What remains constant or consistent?
What is idiosyncratic?
What is missing?
Analytic and interpretive questions (4)
What changes are interrelated?
What changes oppose or harmonise with natural human development or constructed social processes?
What are participant or conceptual rhythms, e.g., cycles through time?
What is the through-line of the study?

**Table 4:** Overview of themes and corresponding changes in levels of health literacy.

Theme	Health Literacy (HL) Level	Subtheme	Categories
Re-engagement with health information	Changes in functional HL	Qualities of communicator Forum/methods	Engaging Supportive Multiple methods used
Increased understanding of risk and protective factors	Changes in functional HL		Food and nutrition Exercise Medication and treatments Side-effects of medication Cholesterol Blood Pressure
Changes in interactions with healthcare providers (HCP)	Changes in interactive HL	More at ease in communicating with the HCP Reinforcement/reassurances	Increased knowledge and therefore confidence
Enhanced psychological insights	Changes in critical HL	Increased insights of personal control limits and opportunities	Self-efficacy and confidence Dealing with stress
		Emotional issues Facilitators of motivation	Anxiety/fear Peer comparisons
Understanding the broader determinants of health changes in critical health literacy	Changes in critical HL	Sharing information Infrastructures to support health	Safe access—walkways, cycling Food manufacturing/culture Government lobbying

## Appendix A. Interview Guide

1. How would you describe your experience of taking part in the programme (a structured CVD risk reduction and health promotion course)?
2. Can you tell me about how you have been getting information about your health/health issues in general since we last met?
3. How has your understanding changed in relation to your own health situation/relation to health issues in general?
4. Have you learnt anything that makes it easier to make judgements on what information is useful and what is not? How have you done this?
5. Tell me about how you have used any information from the course? What has helped/hindered this process?
6. Can you tell me about your understanding of health and well-being issues in general?
7. If it were possible to make any changes in your own neighbourhood, what might you do to make it more health promoting for yourself/your community?
8. Can you tell me what changes you see or feel in yourself as a result of participating in the programme?
